# Supplementary material for: Emulating short-term synaptic dynamics with memristive devices
Source: Sci Rep. 2016 Jan 4;6:18639. doi: 10.1038/srep18639 (PMC4698662; doi:10.1038/srep18639)
Supplement: Supplementary Information [file srep18639-s1.pdf]

Supplementary Information

Emulating short-term synaptic dynamics with memristive devices

Radu Berdan, Eleni Vasilaki, Ali Khiat, Giacomo Indiveri, Alexandru Serb, Themistoklis Prodromakis

Characterization of singe TiO_2 memristors

All stand-alone TiO_2 based memristors were electrically evaluated on-wafer with a Keithley SCS-4200 Semiconductor Characterization Suite. The probe/point contacts to the top (TE) and bottom electrodes (BE) of the devices under test were realized through a pair of Wentworth probe-needles, on a Wentworth Laboratories AVT 702 semi-automatic prober. The current-voltage characteristics of all tested devices were studied by DC voltage sweeps in sequence of 0V to positive voltage and again to 0V, followed by the same procedure in opposite polarity for testing the bipolar behavior and in sequence of 0V to positive voltage and return to 0V for testing unipolar switching modalities. In all voltage sweep evaluations the BE were grounded while distinct voltage biases were applied on the TE of the cells.

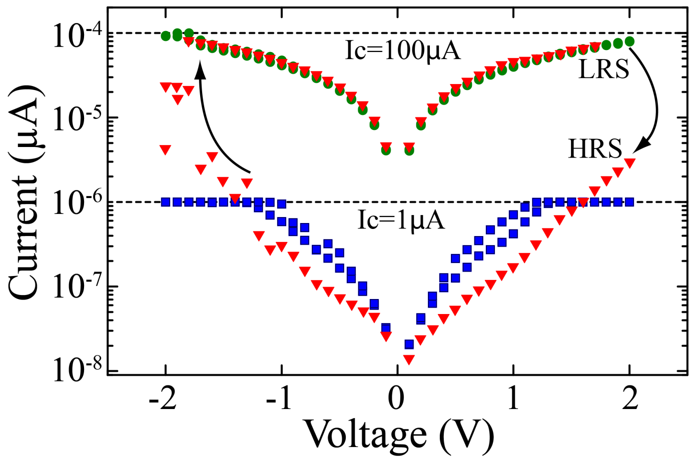

Figure S1 Electrical characteristics of pristine and electroformed TiO_2 memristors. The compliance current was increased from 1μA to 100μA after which bipolar switching could be reproduced within a ±2V biasing range.

All pristine (as-fabricated) devices are initially at a high-resistive state (HRS) as shown in Figure S1. In order to operate the devices as memristors, an initial electroforming step[1,2] was employed that elaborates a soft-breakdown mechanism[3]. This step creates some randomly oriented defects of high conductance within the TiO_2 film that facilitates the establishment of distinct current percolation filaments within the device's core under appropriate biasing conditions. Current compliance plays a dominant role in the electroforming procedure. The voltage sweeps shown in Figure S1 were acquired with the current compliance being initially set at 1μA (HRS) and was then increased to 100μA, allowing the device to acquire a two orders of magnitude lower resistive state (LRS). After this initial testing that ensured all devices are functional, the compliance current was maintained at 100μA throughout

all experiments, with the memory state of the device been allowed to toggle between these two bounding states R_{OFF} and R_{ON}.

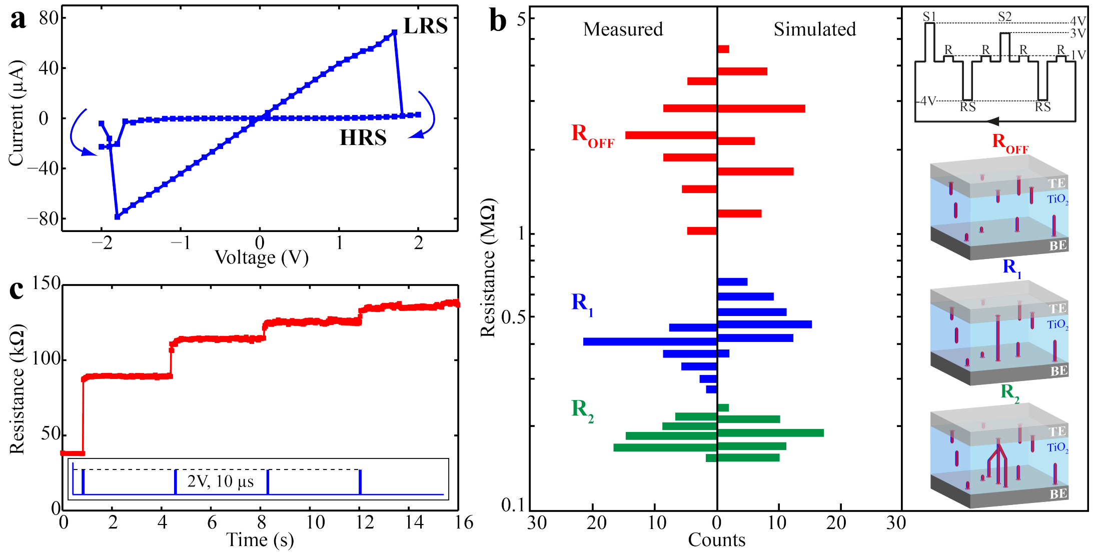

Figure S2 Electrical characteristics of our memristor prototypes. Shown are: a) pinched hysteresis I-V trend that indicates a memristor signature, b) continuous cycling (200 cycles) between three resistive states with measured and simulated response according to a filamentary formation model as shown next to each corresponding state and c) demonstration of the intrinsic accumulating non-linear response of our prototypes when programmed with voltage pulses of fixed amplitude (inset).

After electroforming, the devices are originally in a LRS and a HRS can be achieved as the sweeping voltage bias approaches a set value Vset = 1.7V. Reversing the voltage polarity, the device switches onto a LRS at V_{reset1} = -1.8V (Figure S2a). This action describes completely the bipolar behavior. The pinched hysteresis curve obtained is a clear fingerprint of a memristor[4,5].

Figure S2b demonstrates three resistive states that were obtained from 50 repeated pulsing sequences, of 10µs pulse widths, as described in the corresponding figure inset. The multi-state capacity of our memristor is modeled in this case via a random circuit breaker network model[6-8], as illustrated in the corresponding inset schematics of Figure S2b to simulate the effect of local conductance changes within the active TiO_2 core on the overall conductance of the solid-state device. Considering that a SET potential will facilitate some local modification of the active material in the form of a conductive filament that in turn will result in a state modulation, we represent this change by altering some of the branch resistances to higher conductance values (colored lines). The number of filaments in R_1 and R_2 as well as the corresponding values for the low and high resistive branches is arbitrarily selected for matching the average measured resistive states across all cycles. The experimental and simulated results employed in the RCB model are in close correlation, validating the notion that the attained resistive states are due to filamentary formations/disruptions.

Figure S2c depicts the non-linear accumulating nature of our TiO_2 memristors when subsequent identical voltage pulses have a decreasing effect on the modulation of the effective resistance of our prototypes. This programming method was also

employed, when necessary, to set/reset our devices at intermediate resistive states throughout our experiments.

Short term plasticity characterization

In order to reliably characterize the state-retention exhibited in our memristors, an NXP mBED based test-bed was designed and implemented (Figure S3a). The NXP mBED is a versatile and powerful multipurpose microcontroller capable of reading and outputting analogue voltages in the range of 0 to 3.3 V with 10 bit precision but also outputting digital voltages of 0 (LOW) or 3.3 V (HIGH) for control purposes. The microcontroller was programmed in C.

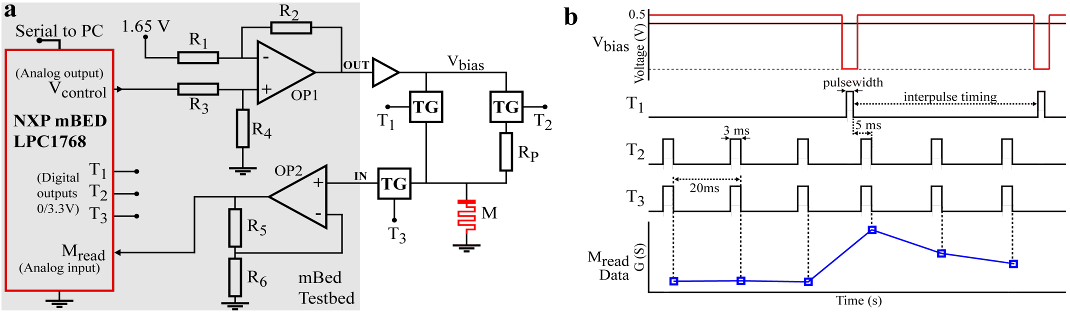

Figure S3 Custom instrumentation test-bed employed for exploiting the metastable characteristics of our TiO_2 based memristors. Shown are: a) circuit schematic and b) Illustration of pulsing pattern to obtain data on transient conductance dynamics.

The following features were exploited in the instrumentation circuit illustrated in Figure S3a: 12 bit DAC at $V_{control}$, 12 bit ADC at M_{read} and digital output pins at T_1,T_2 and T_3. Transmission gates were implemented as ns transition electronic switches in order to obtain sharp voltage pulses applied to our device under test (DUT), denoted here as M. OP1 is a precision op-amp in a voltage subtractor configuration that maps the limited range of output voltages at $V_{control}$ of 0 to 3.3 V into -10 V to +10 V at V_{bias}. Hence a programming pulse can be applied by first setting V_{bias} to the required pulse amplitude value (through setting the $V_{control}$ output to the corresponding voltage) and then closing the analogue switch controlled by T_1 for a set amount of time, which will correspond to the required pulse width. Reading the current resistance value of the device is performed by setting V_{bias} to 0.5 V and closing switches T_2 and T_3 (while keeping T_1 open). The voltage drop across memristor M is amplified by the OP2 configuration (gain of 6) in order to bring the voltage range that can occur across the memristor from 0-0.5 V to 0-3 V and hence utilize the full input range of the M_{read} ADC. The resistance value is then extrapolated by taking into account the reading resistance R_P. An illustration of the pulsing sequence utilized to obtain the data shown in Figure 2 is illustrated in Figure S3b, with adjacent pulse timings included in the read sequence. This circuit was implemented via discrete components: OP1 and OP2 are part of a dual precision op-amp TL5580, transmission gates are ADG452B in a quad package, R_P=470kΩ and the buffer is BUF634. The OP1 and OP2 configurations both have a gain of 6 V/V.

Sequence detector experiment

The circuit shown in Figure S4 was designed to facilitate the imitation of two pre-synaptic spiking potentials through one static (R_S) and one dynamic (M) memristive synapse connected to the same post-synaptic neuron (neuron's schematic appears

on Figure S6). The functionality of the circuit in Figure S3a was maintained in this circuit so that the short-term dynamics of our memristors (dynamic synapses) could be recorded during the experiment.

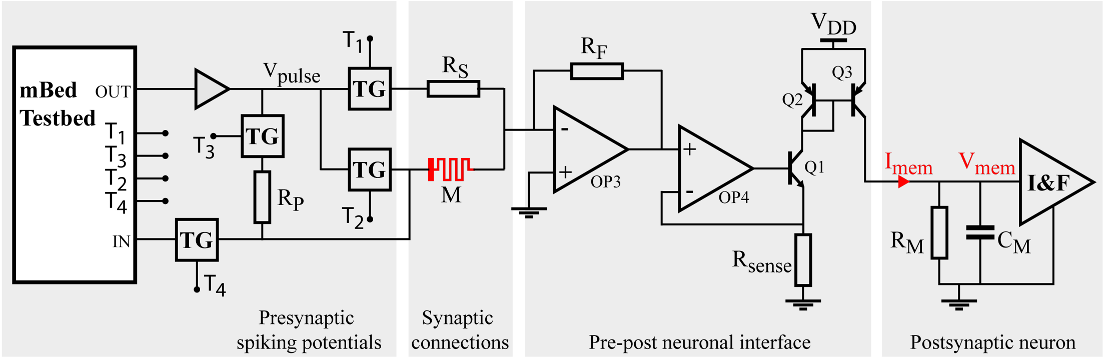

Figure S4 Circuit schematic of the sequence detector experiment, incorporating a static resistor and a ReRAM dynamic synapse, accordingly denoted as R_S and M.

During this experiment, M varied around a 180kΩ average and the success of the experiment depended on the response of the memrisive synapse to the temporal stimulus. This was found to be mostly facilitating, with the contribution to the accumulation of the membrane potential being increasingly larger with each corresponding presynaptic spike. In addition, we have included an interface between the control system (presynaptic neurons) and the postsynaptic exponential Integrate and Fire (eI&F) neuron to isolate the memristor from a potentially disruptive spike of the membrane voltage and also to introduce a degree of flexibility (amplification).

The presynaptic potentials are applied on the R_S and M synapses sequentially, as illustrated in Figure S4. The weight (conductance) of each synapse sets the current through each branch at the input of OP3 that add up at the inverting pin. The output voltage of OP3 is fed to OP4, which along with R_{sense} and the current mirror translate this voltage into a proportional current, which is then fed in the membrane of the eI&F neuron represented by the leaky integrator formed by R_M and C_M.

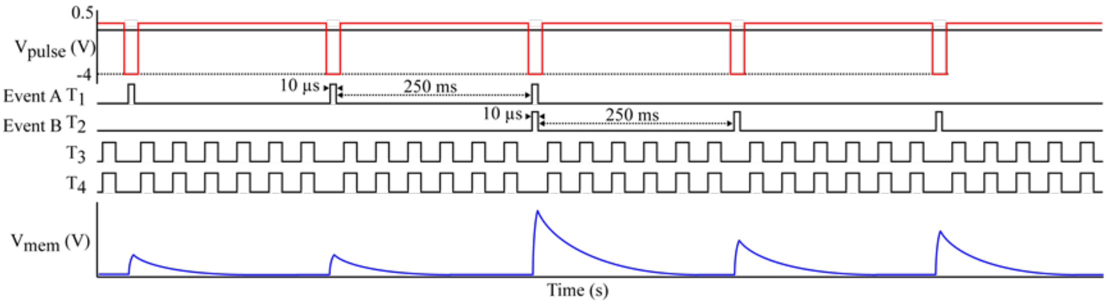

Figure S5 Illustration of one pulsing scheme for Event AB case of the sequence detector experiment.

The pulsing scheme utilized to achieve the experimental data shown in Figure 4, is shown in Figure S5 for the Event AB case. Voltage pulses of set amplitude of -4V are applied to the static resistor R_S by closing the T1 transmission gate then on the dynamic synapse represented by M by closing the T_2 transmission gate. In between biasing pulses (interpulse time of 250ms), the mBed samples the DUT's conductance.

The experiment was repeated 40 times for each case (A before B and B before A) with a recovery time of 20 seconds between each test, to allow for the memristive synapse to fully restore its original memory state.

A discrete-based Integrate & Fire neuron

The neuron circuit of Figure S6 represents the hardware implementation of the adaptive exponential integrate-and-fire model[9] used in this work. The circuit, implemented using discrete analog electronic components, is inspired by the original Very Large Scale Integration (VLSI) Silicon Neuron[10], and includes elements of the adaptive integrate-and-fire circuit silicon neuron circuit described in ref [11]. Specifically, the passive leak behavior of the neuron is implemented using a passive RC circuit; active filter circuits reproduce the dynamics of the voltage dependent Sodium and Potassium conductances; and the exponential dependence on the membrane potential is implemented by means of a bipolar transistor. As current is injected into the circuit membrane capacitor C_M the membrane voltage V_{mem} rises following the RC dynamics. As V_{mem} crosses a threshold voltage V_{REF}, both Sodium and Potassium active filters are activated. After a short delay the Sodium filter drive a PNP bipolar transistor. As the bipolar transistor is activated, a positive-feedback current is injected in the membrane capacitor, and an axon potential is quickly generated. As the Potassium filter increases its output amplitude, after a longer delay, an NPN bipolar transistor is activated to reset the membrane potential. As V_{mem} falls below V_{REF} both filters are switched off and the passive integration of the input current starts a new cycle.

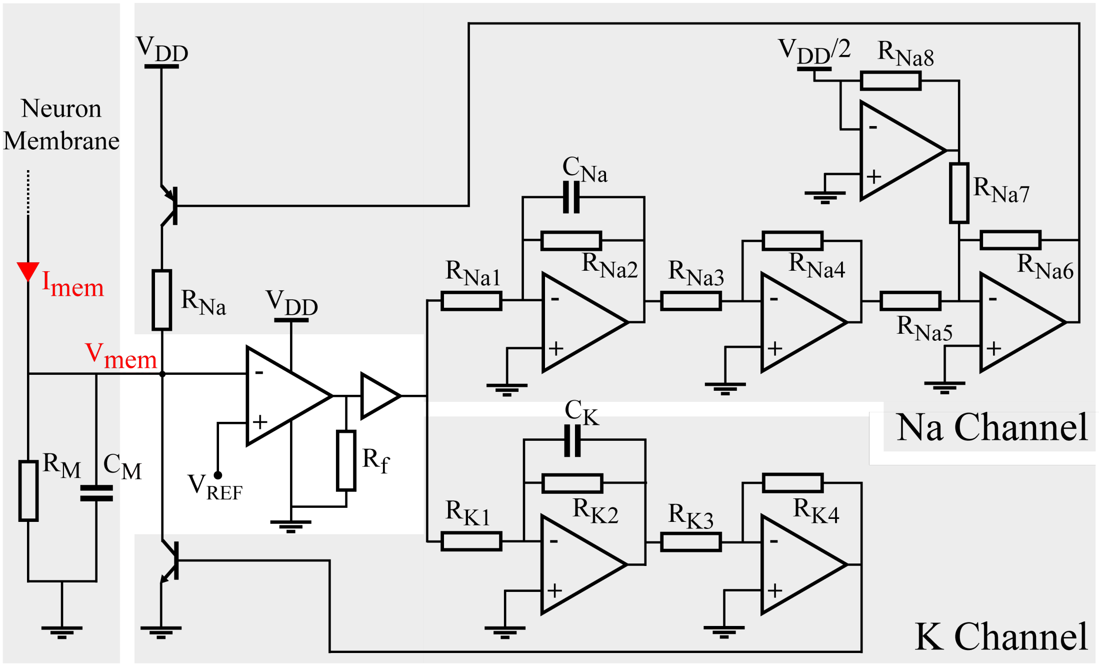

Figure S6 Exponential Integrate & Fire Neuron circuit schematic using discrete components.

Short-Term Facilitation and Depression model

The running value of the synaptic conductance *G* changes on the short-term time scale as a function of the firing activity history of the presynaptic neuron[12,13]. According to the model, the variable *G* depends on the fraction of the available resources $u\,r$ of the synapse:

$$G = \ A\ u\ r, \tag{1}$$

where *A is* a scaling factor and *u* , *r* are defined at the arrival *n* + 1 by the following iterative expressions:

$$\begin{aligned} r_{n+1} &= (r_n(1-u)-1)\exp(-\Delta t_n/\tau_{rec})+1, \\ u_{n+1} &= u_n(1-U)\exp(-\Delta t_n/\tau_{facil})+U, \end{aligned} \tag{2}$$

with τ_{rec}, τ_{facil} representing the time constants involved in these processes, U the release probability of the synapse, r_n the value of *r* at the arrival of the *n*th spike, u_n the value of u immediately after the arrival of the *n*th spike, and Δt_n the time elapsed from the n-th to the $n+1$ spike. Both short-term depression and facilitation are captured by the same set of equations, depending on the parameters used. In general, facilitating synapses have low release probability, low τ_{rec} and high τ_{facil}, while depressing synapses have high release probability, high τ_{rec} and low τ_{facil}. To reduce the number of free parameters, we set *A* equal to the maximum conductance of the sample and for the behaviour that appears to be facilitating (i.e. increasing conductance values), we fix τ_{rec} = 1*ms* (an arbitrary small value), while for the behaviour that we have termed “saturating” (i.e. decreasing conductance values), we fix τ_{facil} = 1*ms*. We defined the error function as the summed normalised difference between the memristive measurements and the model, and make use of the Matlab optimisation toolbox. We then fit two free parameters in each case, resulting for the case of the facilitating memristive synapse U = 0.82 and τ_{facil} = 6250*ms* and for the case of the saturating memristive synapse U = 0.96 and τ_{rec} = 198*ms*. We would like to underline, however, that the saturating synapse fitted when allowing three free parameters will result combinations of τ_{facil} and τ_{rec} that are compatible with both facilitating and depressing behaviour, hence we cannot robustly determine the nature of the synapse via fitting. The underlining physical mechanisms of the memristor indicated in the main text point to the direction of a facilitating (saturating) behaviour.

Table 1 Fitted parameters for our model.

Symbol	Description	Value
U_S	Release probability, for saturating synapses	0.96
U_F	Release probability, for facilitating synapses	0.82
$\tau_{rec\ S}$	Recovery from depression, for *saturating* synapses	198 *ms*
$\tau_{rec\ F}$	Recovery from depression, for *facilitating* synapses	1 *ms*
$\tau_{facil\ S}$	Recovery from facilitation, for *saturating* synapses	1 *ms*
$\tau_{facil\ F}$	Recovery from facilitation, for *facilitating* synapses	6250 *ms*
A_S	Scaling factor for *saturating* synapses	2.16 μS
A_F	Scaling factor for *facilitating* synapses	2.165 μS

Neuron Membrane Potential Model

To extrapolate the contributions of a voltage pulse through our volatile memristor in Fig. 2, a neuron's membrane was modelled as a capacitor in parallel with a static resistor, which is supplied by a current source during an input stimulus with current proportional to the conductance drift of our device and the amplitude of the pulse. These quantities are illustrated in Figure S7. The conductance drift was approximated as a linear drift from C_0, the conductance measured right before a pulse, and C_{tp}, the conductance measured right after the application of the pulse.

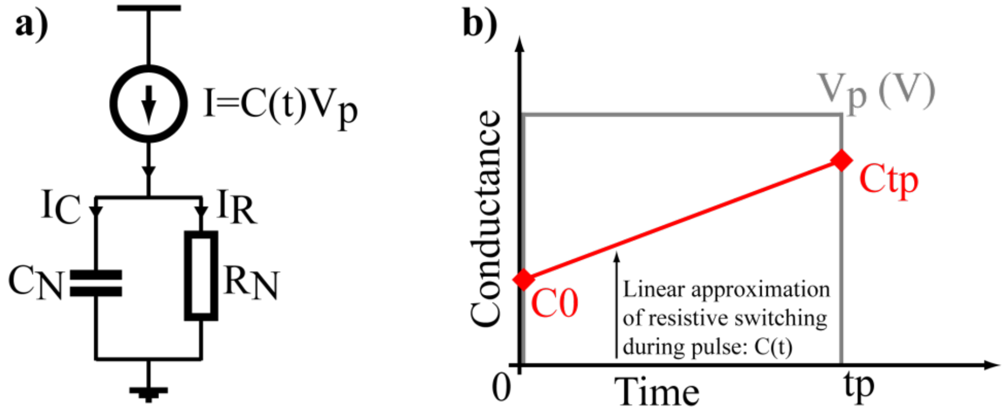

Figure S7: a) Membrane potential equivalent circuit. b) Illustration of conductance drift during one input pulse with a linear function as an approximation of the resistive switch, *Vp* is the pulse amplitude and *tp* is the pulse width.

Hence:

$$\boldsymbol{C(t) = C0 + \frac{(Ctp - C0)t}{tp}} \tag{8}$$

During an input pulse, the membrane potential *Vmem* follows:

$$\boldsymbol{Vmem(t) = R_N * C(t) * Vp - R_N C_N \frac{dVmem(t)}{dt}} \quad (9)$$

Which has the solution:

$$\boldsymbol{Vmem(t) = R_N * C_0 * Vp - R_N\tau \frac{(Ctp - C_0)Vp}{tp} + R_N \frac{(Ctp - C_0)V\square}{tp} t + K * e^{\frac{-t}{\tau}} + Vmem(0)} \quad (10)$$

Where:

$$\boldsymbol{K = \frac{R_N\tau(Ctp - C_0)Vp}{tp} - VpR_N C_0 + Vmem(0)} \quad (11)$$

$$\boldsymbol{\tau = R_N C_N} \quad (12)$$

$$Vmem(0) = membrane\ potential\ at\ t = 0\ (onset\ of\ pulse)$$

In between pulses, the membrane potential follows:

$$\boldsymbol{Vmem(t) = Vmem(0) * e^{\frac{-t}{\tau}}} \quad (13)$$

Metastable effects present over a wide conductance range

To support our argument that metastable effects in TiO_2 memristors are apparent across a wide conductance range and for various stimuli, as depicted in the model presented in this work (Figure 1f), Figure S8 illustrates STP-F and STP-S events across distinct conductance levels. These measured results were acquired with different voltage pulsing schemes. The corresponding STP-F or STP-S response becomes more apparent for relatively large pulse amplitudes, with the risk however to result into a long-term non-volatile transition.

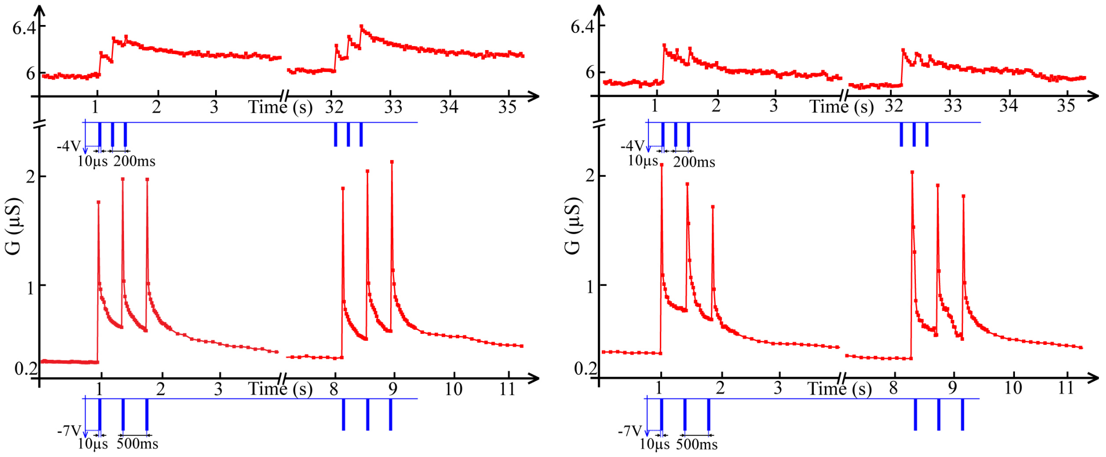

Figure S8 Transient response of a single memristor while pulsed with similar stimuli. Both short-term facilitation and depression is demonstrated across a wide conductance range, proving the versatility of this effect.

Probabilistic expression of STP-F and STP-S events

A single device was subjected to a train of 3 consecutive voltage pulses of -4V, 10µs wide and inter-pulse interval t_{int}=400ms. This sequence was repeated 600 times with a recovery interval between sequences t_{rec}=10s, to allow the conductance to return to its original state. The two types of STP-F and STP-S events were discriminated, as shown in Figure S9, with respect to the initial conductance at which each 3-pulse sequence was applied. Our results indicate a clear preference for facilitating responses, in accordance with the energetically favored trend illustrated in Figure 1b. It is interesting to note that approximately a third of the counted events follow a saturating response, with the STP-S distribution peaks appearing at slightly elevated initial conductance levels than the STP-F ones. By observing the probability of STP-F and STP-S events occurrence within distinct initial conductance bins (insets of Figure S9), it is evident that facilitating (saturating) events are more likely to occur when G_0 is relatively low (high); illustrating a strong probabilistic switching nature.

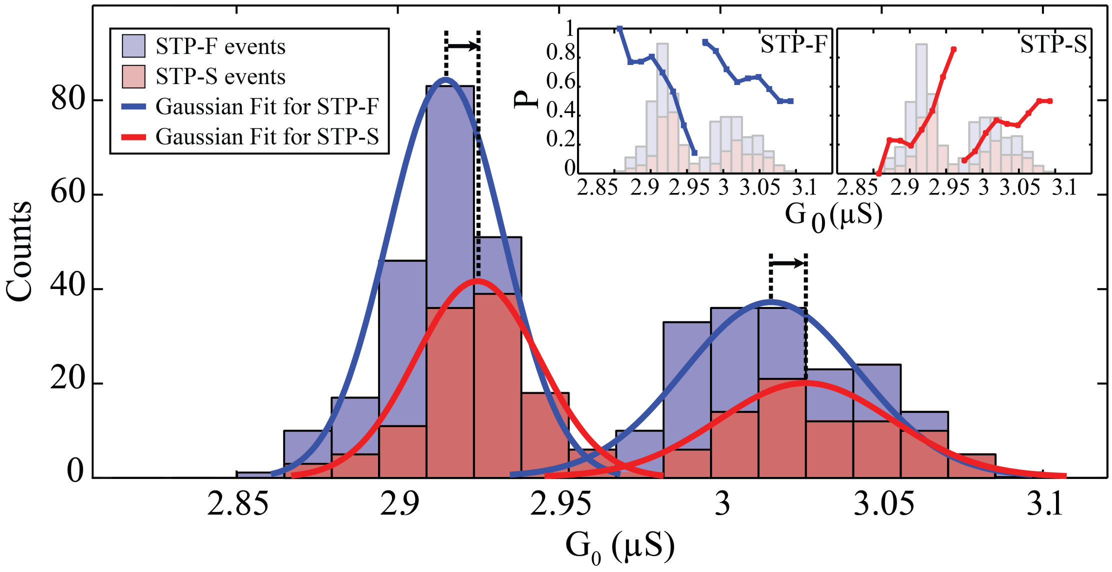

Figure S9 Probabilistic expression of STP-F and STP-S events. Shown are 600 memristor plasticity events all acquired from a single device, invoked with the same stimuli, that were categorized as STP-F and STP-F with Gaussian fittings for both demonstrating a probabilistic response. The probability of occurrence of STP-F and STP-S events is shown in the corresponding insets.

Interpulse effect experiment methodology

A clear correlation between the state restoration and interpulse timing was found. Our device was stimulated by a two pulse pattern with variable inter-pulse timings. For each pulse rate, the measurements were repeated three times with a two-minute resting time in between stimuli sets over which the decay rate of the memristor was recorded. All three events were then averaged and normalized conductance decay curves for each stimulating rate were plotted in Figure S10 a-f for inter-pulse timings of 200, 100, 80, 60, 40 and 20 ms relating to pulse rates of 5 to 50 Hz. The inset in each figure shows the inter-spike time of the respective stimulus.

The conductance decay after the last stimulating pulse can be modeled by a stretch exponential function, listed as equation 16. The choice of this particular function was supported by previous uses to describe relaxation of disordered material structures[14].

$$\frac{\Delta G}{G_0} = scale \cdot e^{-\left(\frac{t}{\tau}\right)^{\beta}} + C_{settle} \tag{16}$$

Where G_0 is the initial conductance point and β was held constant as a material dependent parameter. For this experiment, $\beta = 0.215$.

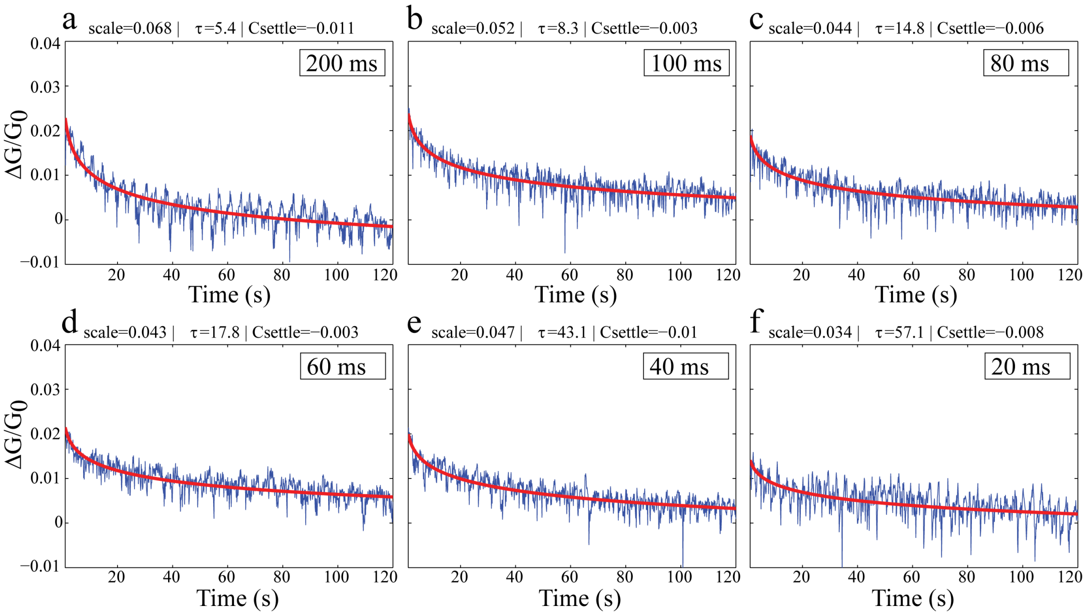

Figure S10 Short-term plasticity under a pulsing pattern of two pulses of -4V, 10 µs and interpulse times of: a) 200 ms, b) 100 ms, c) 80 ms, d) 60 ms, e) 40ms and f) 20 ms. Blue line are measured data, red lines are fitting curves via equation 16. Title of each sub-figure contains the parameters of the employed fittings.

The extracted parameters of the fittings in Figure S10 were plotted with respect to interpulse timing in Figure 3a showing a clear trend. The pulsing rate is proportional to the time constant of the resistive state decay indicating a shift towards long term plasticity.

Sequence detector control experiment / Coincidence detector

In order to benchmark the reliability of our results, we devised a *sequence-detector* control experiment (Figure S11) in which we have replaced the memristive synapse with static components: a 180kΩ resistor R_{SM} (based on the average resistive values encountered over the course of our initial experiments) in parallel with a capacitor C_{SM}=2.2pF (measured parasitic capacitance of the employed package). This control group is designed to show that the non-linear response of the memristive synapse to the spike input is crucial for the sequence detector. Similarly to the original experiment described in Figures 4 and S4, we repeated the test for 40 times for each event case (AB or BA). The corresponding circuit schematic is shown in Figure S11. Setting R_M= 10MΩ and C_M=20nF corresponds into mimicking a neuron's membrane with a time constant of 50ms, and R_S is 560 kΩ. Under these circumstances, the discrete neuron fires at low rates for both AB and BA sequences of events due to the

fact that each pre-synaptic pulse contributes equally to the membrane potential through both static synapses, making AB and BA sequences undistinguishable. The non-zero spiking rate is due to the low neuron membrane threshold coupled with the inherent noise of the system. The STP behavior of the memristor is thus essential to the differentiation in between the temporal position of the stimuli.

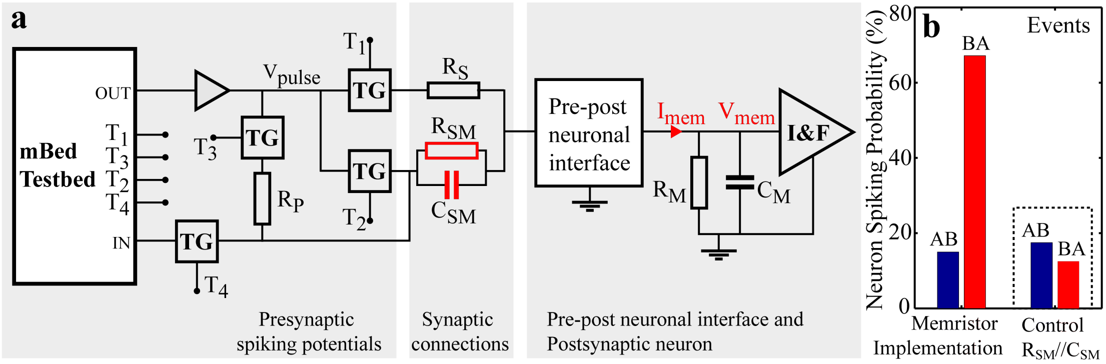

Figure S11 Control sequence detector experiment. Shown are: (a) corresponding circuit schematic in which the memristive synapse is replaced by static components denoted as R_M and C_M and (b) neuron spiking probability for both sequence of events.

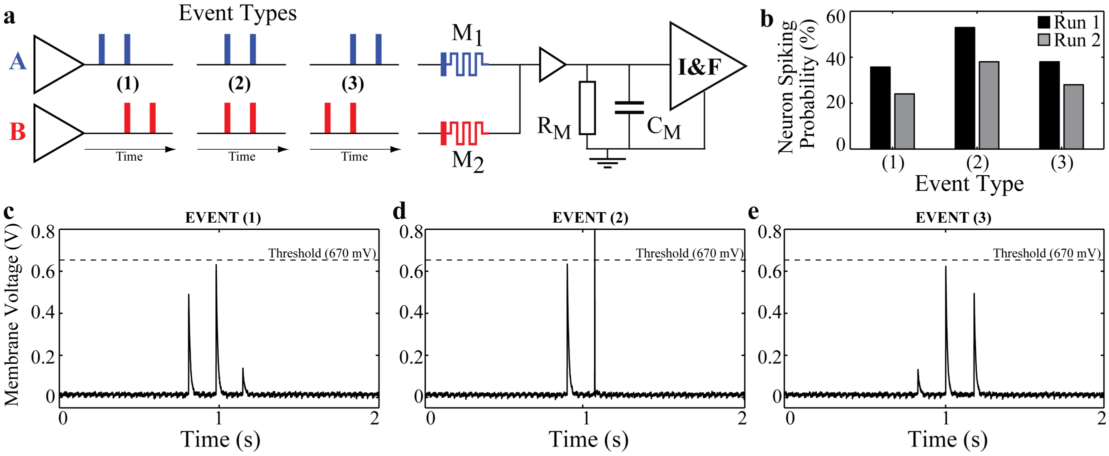

Figure S12 Demonstration of sequence detector with two memristive synapses. a) Illustration of the coincidence detector circuit, b) Neuron spiking probability for all events in two different runs: during Run 1 the membrane threshold was set to 670mV, during Run 2 the threshold was raised to 675 mV Example of measured transient response of the neuron membrane potential for Event (1) (c), Event (2) (d) and Event (3) (e).

The experiment was performed by applying each type of Event ((1) first, followed by (2) and (3)) 10 times each in sequence, with a 5 second resting time in between each Event, then the whole set was repeated 7 times. The number of output spikes was counted and the total for each type of Event was normalised by the number of total common input pulses (1 per each Event in the case of Event (1) and (3), and 2 in the case of Event (2)). The results are plotted in Figure S12b showing an increased spiking probability of the post-synaptic neuron in the case of Event (2) compared to Events (1) and (3), marginally distinguishing the concurrence of input signals A and B due to device variability.

References

1. Buonomano, D. V. D. & Merzenich, M. M. M. Temporal information transformed into a spatial code by a neural network with realistic properties. *Science* **267,** 1028–1030 (1995).
2. Joshua Yang, J. *et al.* The mechanism of electroforming of metal oxide memristive switches. *Nanotechnology* **20,** 215201 (2009).
3. Alam, M. A., Weir, B. E. & Silverman, P. J. A study of soft and hard breakdown - Part I: Analysis of statistical percolation conductance. *IEEE Trans. Electron Devices* **49,** 232–238 (2002).
4. Chua, L. O. Memristor-The missing circuit element. *Circuit Theory, IEEE Transactions on* **18,** 507–519 (1971).
5. Kim, H., Sah, M. P. & Adhikari, S. P. Pinched Hysteresis Loops is the Fingerprint of Memristive Devices. *arXiv.org* **cond-mat.mes-hall,** (2012).
6. Lee, S. B. *et al.* Interface-modified random circuit breaker network model applicable to both bipolar and unipolar resistance switching. *Appl. Phys. Lett.* **98,** 033502 (2011).
7. Shihong, M. W., Prodromakis, T., Salaoru, I. & Toumazou, C. Modelling of Current Percolation Channels in Emerging Resistive Switching Elements. *arXiv.org* **cond-mat.mes-hall,** (2012).
8. Chae, S. C. *et al.* Random Circuit Breaker Network Model for Unipolar Resistance Switching. *Adv. Mater.* **20,** 1154–1159 (2008).
9. Brette, R. Adaptive Exponential Integrate-and-Fire Model as an Effective Description of Neuronal Activity. *Journal of Neurophysiology* **94,** 3637–3642 (2005).
10. Mahowald, M. M. & Douglas, R. R. A silicon neuron. *Nature* **354,** 515–518 (1991).
11. Indiveri, G. G. *et al.* Neuromorphic silicon neuron circuits. *Front Neurosci* **5,** 73–73 (2011).
12. Markram, H. H. & Tsodyks, M. M. Redistribution of synaptic efficacy between neocortical pyramidal neurons. *Nature* **382,** 807–810 (1996).
13. Tsodyks, M., Pawelzik, K. & Markram, H. Neural networks with dynamic synapses. *Neural computation* **10,** 821–835 (1998).
14. Sturman, B., Podivilov, E. & Gorkunov, M. Origin of Stretched Exponential Relaxation for Hopping-Transport Models. *Phys. Rev. Lett.* **91,** 176602 (2003).
